# Diagnostic Testing of Pediatric Fevers: Meta-Analysis of 13 National Surveys Assessing Influences of Malaria Endemicity and Source of Care on Test Uptake for Febrile Children under Five Years

**DOI:** 10.1371/journal.pone.0095483

**Published:** 2014-04-18

**Authors:** Emily White Johansson, Peter W. Gething, Helena Hildenwall, Bonnie Mappin, Max Petzold, Stefan Swartling Peterson, Katarina Ekholm Selling

**Affiliations:** 1 International Maternal and Child Health, Department of Women’s and Children’s Health, Uppsala University, Uppsala, Sweden; 2 Spatial Ecology and Epidemiology Group, Department of Zoology, University of Oxford, Oxford, United Kingdom; 3 Global Health, Department of Public Health Sciences, Karolinska Institutet, Stockholm, Sweden; 4 Center for Applied Biostatistics, University of Gothenburg, Gothenburg, Sweden; 5 School of Public Health, College of Health Sciences, Makerere University, Kampala, Uganda; Tulane University School of Public Health and Tropical Medicine, United States of America

## Abstract

**Background:**

In 2010, the World Health Organization revised guidelines to recommend diagnosis of all suspected malaria cases prior to treatment. There has been no systematic assessment of malaria test uptake for pediatric fevers at the population level as countries start implementing guidelines. We examined test use for pediatric fevers in relation to malaria endemicity and treatment-seeking behavior in multiple sub-Saharan African countries in initial years of implementation.

**Methods and Findings:**

We compiled data from national population-based surveys reporting fever prevalence, care-seeking and diagnostic use for children under five years in 13 sub-Saharan African countries in 2009–2011/12 (n = 105,791). Mixed-effects logistic regression models quantified the influence of source of care and malaria endemicity on test use after adjusting for socioeconomic covariates. Results were stratified by malaria endemicity categories: low (*Pf*PR_2–10_<5%), moderate (*Pf*PR_2–10_ 5–40%), high (*Pf*PR_2–10_>40%). Among febrile under-fives surveyed, 16.9% (95% CI: 11.8%–21.9%) were tested. Compared to hospitals, febrile children attending non-hospital sources (OR: 0.62, 95% CI: 0.56–0.69) and community health workers (OR: 0.31, 95% CI: 0.23–0.43) were less often tested. Febrile children in high-risk areas had reduced odds of testing compared to low-risk settings (OR: 0.51, 95% CI: 0.42–0.62). Febrile children in least poor households were more often tested than in poorest (OR: 1.63, 95% CI: 1.39–1.91), as were children with better-educated mothers compared to least educated (OR: 1.33, 95% CI: 1.16–1.54).

**Conclusions:**

Diagnostic testing of pediatric fevers was low and inequitable at the outset of new guidelines. Greater testing is needed at lower or less formal sources where pediatric fevers are commonly managed, particularly to reach the poorest. Lower test uptake in high-risk settings merits further investigation given potential implications for diagnostic scale-up in these areas. Findings could inform continued implementation of new guidelines to improve access to and equity in point-of-care diagnostics use for pediatric fevers.

## Introduction

For many years presumptive anti-malarial treatment for febrile children was promoted in malaria-endemic African countries due to lack of diagnostic tools, resulting in widespread malaria over-diagnosis [1], non-rational use of anti-malarial drugs [2], and poor quality treatment of other fever causes [3]. In 2010, however, the World Health Organization (WHO) revised guidelines to recommend diagnosis of all suspected malaria cases before starting treatment based on expert recommendations and increasing availability of malaria rapid diagnostic tests (mRDTs) [4]. Higher anti-malarial drug costs also drive the need for better precision in treatment [5].

The shift from presumptive treatment of febrile children to test-based case management has great potential to improve malaria surveillance, rational drug use and appropriate management of febrile illnesses [6]. By 2010, 37 African countries had a malaria diagnosis policy for all age groups and programs are now investing in wide-scale mRDT provision [7].

Despite this investment, evidence to date regarding malaria diagnostic test practices in sub-Saharan Africa is largely derived from adherence studies in limited health facility settings [8]–[22], or from qualitative interviews of health workers with limited external validity [23]–[27]. There has been no large-scale, systematic assessment of malaria test use at the population level as countries start scaling up diagnostics in line with revised international guidelines. There is also a limited understanding of factors associated with diagnostic test uptake for pediatric fevers, particularly in relation to patterns of malaria endemicity and treatment seeking behavior that may vary substantially within countries.

Malaria transmission intensity has long been known to influence the management of acute febrile illnesses in children, resulting in common malaria over-diagnosis in malaria-endemic settings [28]. Malaria endemicity has also been hypothesized to specifically affect malaria diagnostic testing practices [29] similar to how local disease epidemiology influences diagnostic use for pediatric infections in high-income countries [30]. Yet, there is currently limited understanding of this key relationship between local malaria epidemiology and the use of diagnostic tests to confirm malaria infection.

Similarly, it is likely that treatment-seeking behavior greatly influences whether a febrile child gets tested for malaria. Microscopy has historically been concentrated at hospitals and higher-level health facilities [31], and initial mRDT implementation has also targeted formal health system sources [6]. Yet, most pediatric febrile illnesses are managed at home or in community settings where diagnostic tests are near absent [32]. Recent research indicates that the largest contributor to reduced systems effectiveness of malaria case management in Zambia is where care was sought for the sick child [33]. Individual characteristics, notably maternal education, may also affect test use given their well-known role in the uptake of other child survival interventions [34]. Yet, there is no evidence about such factors, nor if these individual influences are conditioned by the child’s residence or malaria risk.

In 2009, Roll Back Malaria (RBM) recommended asking a question on malaria diagnostic test use in national population-based surveys [35]. Since this time, comparable data have been collected in Demographic and Health Surveys (DHS) [36], Multiple Indicator Cluster Surveys (MICS) [37], Malaria Indicator Surveys (MIS) [38] and ACT Watch Household Surveys [39]. We analyzed these new data to assess extent and determinants of malaria diagnostic test use for pediatric fevers in multiple sub-Saharan African countries during initial years of implementing new guidelines. This paper represents an early assessment against which future progress in diagnostic scale-up may be measured.

## Methods

### Data Sources

**Figure 1:**
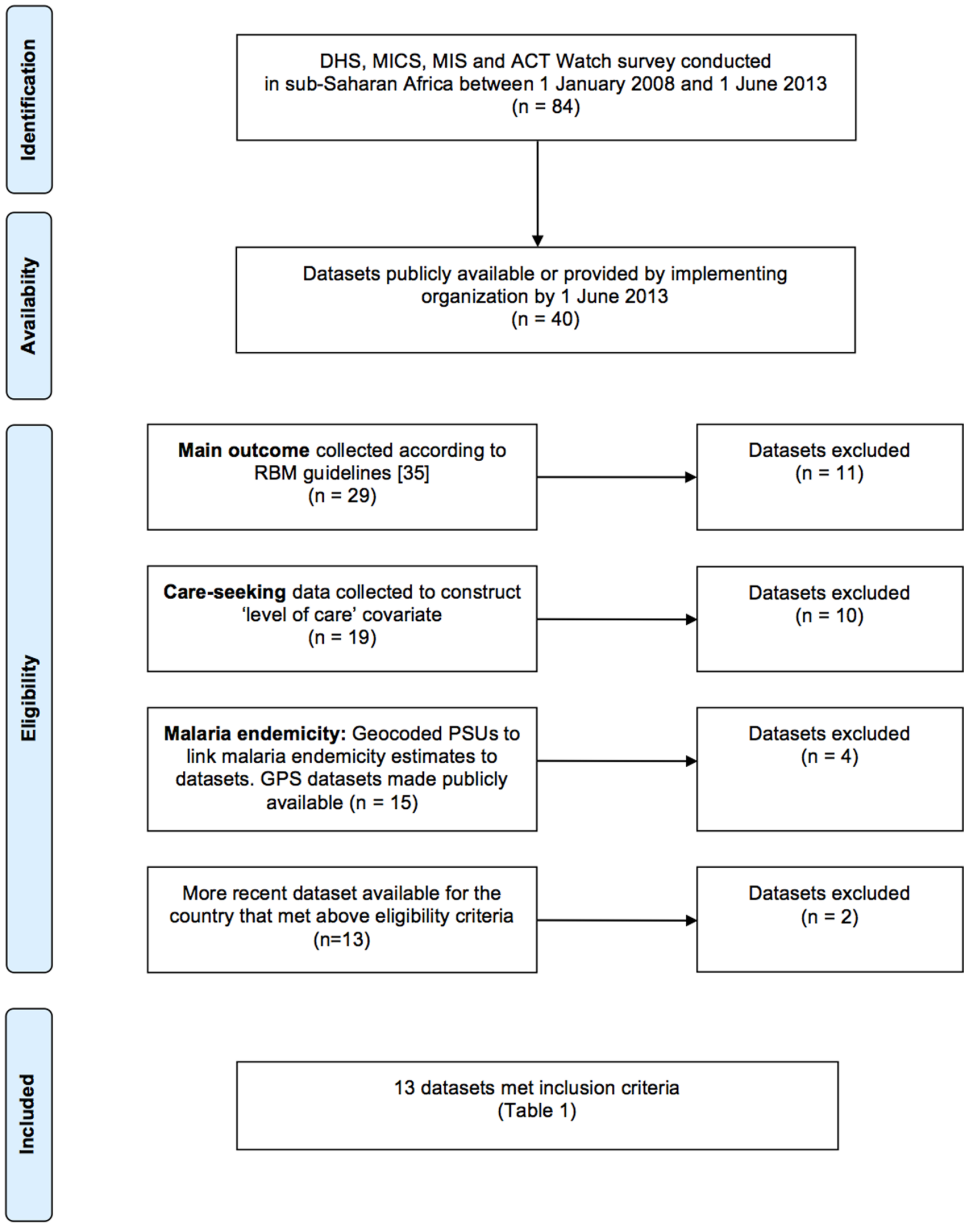
Flow chart of inclusion criteria for study.

National population-based cross-sectional surveys from DHS, MICS, MIS and ACT Watch conducted in sub-Saharan Africa since 2008 were systematically reviewed for inclusion in this study (Figure 1). 84 surveys were conducted in sub-Saharan African countries between 1 January 2008 and 1 June 2013; 40 datasets were publicly available by 1 June 2013 or were made available by the implementing organization. All datasets were included if they measured the outcome according to RBM guidelines [35], and main covariates as described below. 11 surveys did not collect the outcome measure, or data were collected using non-standard methods. 14 surveys did not collect information to measure main covariates (source of care or malaria endemicity). Two datasets were excluded because a more recent survey was available for the country.

13 DHS and MIS met inclusion criteria, which spanned the period 2009–2011/12 (Table 1). Survey methods are described elsewhere [36]. All surveys with one exception were conducted after national policies were changed to recommend parasitological diagnosis for all age groups prior to treatment, although countries were at different stages of operationalizing these policies at the time of survey fieldwork [40]. For this reason, country-level results are included as a supplement to this paper (Table S1).

**Table 1:** Survey information for 13 countries.

Country	Survey	Year	Fieldwork months	*n* PSUs	*n* Under-fives	Percent under-fives withfever (95% CI)^a^		*n* Febrileunder-fives	Percent febrile under-fivestested (95% CI)^b^		Year of nationalpolicy change^c^
Angola	MIS	2011	January-May	240	7,782	34.1	(31.9–36.2)	2,652	25.9	(23.0–28.9)	2010
Burkina Faso	DHS	2010–2011	May-January	574	14,001	20.6	(19.5–21.7)	2,886	5.3	(4.3–6.3)	2009
Burundi	DHS	2010–2011	August-January	376	7,418	30.1	(28.6–31.7)	2,236	27.0	(24.4–29.6)	2007
Lesotho	DHS	2009–2010	October-January	400	3,348	17.2	(15.7–18.8)	577	10.0	(6.7–13.2)	–
Liberia	MIS	2011	September-December	150	2,876	49.2	(46.4–52.1)	1,416	33.3	(28.9–37.7)	2005
Madagascar	MIS	2011	April-May	268	6,377	14.7	(13.0–16.4)	938	6.2	(4.0–8.5)	2006
Malawi	DHS	2010	June-November	849	18,013	34.5	(33.0–36.0)	6,214	17.4	(15.8–19.1)	2011
Nigeria	MIS	2010	October-December	239	5,519	35.4	(32.3–38.6)	1,956	5.4	(4.1–6.8)	2006
Rwanda	DHS	2010–2011	September-March	492	8,605	15.8	(14.8–16.7)	1,355	21.0	(18.5–23.5)	2009
Senegal	DHS	2010–2011	October-April	391	10,893	22.6	(20.8–24.4)	2,463	9.7	(7.8–11.6)	2007
Tanzania	AIS/MIS	2011–2012	December-May	583	8,216	20.4	(18.8–22.0)	1,675	24.9	(21.2–28.7)	2009 (mainland); 2006 (Zanzibar)
Uganda	DHS	2011	June-December	404	7,535	40.4	(38.1–42.7)	3,042	25.9	(23.2–28.6)	1997
Zimbabwe	DHS	2010–2011	September-March	406	5,208	9.7	(8.8–10.7)	506	7.4	(4.9–9.8)	2008
**Total**					**105,791**	**26.5**	**(21.0–32.0)**	**27,916**	**16.9**	**(11.8–21.9)**	

DHS refers to Demographic and Health Survey. MIS refers to Malaria Indicator Survey. AIS refers to AIDS Indicator Survey. PSU refers to primary sampling unit.

a Children less than five years old reportedly having fever in the 2 weeks prior to the interview.

b Febrile children less than five years old reportedly receiving a finger or heel stick for testing.

c [40] Refers to year national policy changed to recommend parasitological diagnosis in patients of all ages prior to treatment.

### Outcome and Explanatory Covariates

Malaria diagnostic test use is measured by asking caregivers of children under five with reported fever in the past two weeks if “At any time during the illness did (name) have blood taken from his/her finger or heel for testing?” This question does not differentiate between diagnostic tests, and is assumed to refer to either microscopy or mRDT.

There were two main covariates: source of care and malaria endemicity. Source of care is measured by asking caregivers of febrile children if they sought advice or treatment for the illness, and if so, from where care was sought. Multiple responses are allowed, and response categories are standardized across countries with some modifications to account for different health system structures. This covariate was categorized as: (1) hospital (2) non-hospital formal medical (3) community health worker (CHW) (4) pharmacy (5) other (6) no care sought. Hospital, CHW, and pharmacy include any such listed response. Non-hospital includes any formal health system source that is not a hospital or CHW, including health centers or posts, outreach or mobile clinics, and private doctors. Some countries include additional sources for this category, such as maternities or municipal clinics. Other includes shops, traditional practitioners, relatives, and non-specified sources. ‘Hospital’ and ‘non-hospital’ categories were further dichotomized into public or private sources to analyze test uptake across different managing authorities.

The questionnaire does not explicitly record where testing occurred, but plausibly happened where care was sought. If the child visited multiple sources (e.g. hospital and pharmacy), it was assumed testing occurred at the highest level attended and the covariate was coded using a hierarchical stepwise approach. We conducted a sensitivity analysis by comparing adjusted odds ratios with a covariate constructed by excluding febrile children visiting both hospital and non-hospital sources. In this approach, 732 febrile children visited multiple sources in 13 countries; 367 were excluded that visited both hospital and non-hospital sources. No significant difference was found between approaches (data not shown).

Malaria Atlas Project estimates of malaria endemicity were included in the model, which are described elsewhere [41]. Briefly, the geographical limits of malaria transmission were estimated using routine reporting data and biological models of transmission-limiting aridity and temperature conditions. Within these limits, parasite prevalence survey data were assembled, geolocated, and used within a Bayesian geostatistical model to interpolate a continuous space-time posterior prediction of age-standardized *Plasmodium falciparum* parasite rate in 2–10 year olds (*Pf*PR_2–10_) for every 5×5-km pixel for the year 2010. Malaria endemicity estimates were linked to survey datasets through geocoded PSUs. All individual observations were assigned their PSU-level malaria risk value, which was then categorized into one of five malaria endemicity classes: malaria free; unstable transmission; and low (*Pf*PR_2–10_<5%), medium (*Pf*PR_2–10_ 5%–40%), and high (*Pf*PR_2–10_>40%) stable endemic transmission.

Socioeconomic covariates associated with child survival intervention uptake were incorporated in the model. These included child’s age and sex, maternal age and education, household wealth and density, and residence [42], [43]. Child’s age was categorized as 0–5, 6–11, 12–23, 24–35, 36–47, 48–59 months. Maternal age was categorized as 15–24, 25–29, 30–34, 35–39, 40–49 years. Maternal education was categorized as no education, primary and at least secondary education attendance. A household wealth index is pre-specified in datasets and described elsewhere [44]. Household density was categorized as 1–4, 5–8, 9–12 and 13 or more household members [45]. Residence was dichotomized as urban or rural.

Among 29,245 febrile children under five surveyed in 13 countries, 300 had missing values for the outcome, 312 for source of care, 752 for malaria endemicity, and one for maternal education. 36 had missing values for two or more variables. Listwise deletion was used to exclude observations with any missing value from the analysis.

### Data Analysis

Mixed-effects logistic regression models were used to quantify the influence of covariates on malaria test use in pooled and individual country datasets. PSUs were nested within country identifiers and normal distribution of the random effects was assumed. Covariates were included as categorical fixed effects nested within PSUs. Crude odds ratios of main covariates (malaria endemicity and source of care) were initially estimated for their effect on the outcome. Main covariates were then included simultaneously in one model and, subsequently, odds ratios were adjusted for the effect of all covariates, as listed above. We tested for an interaction between maternal education and malaria endemicity in the final model, and separately for an equivalent interaction between maternal education and residence. Results were stratified by malaria endemicity categories and separately by residence to examine effect differences across contexts. The level of statistical significance was set to 0.05. National point estimates were tabulated using sample weights pre-specified in datasets, and proportions for the pooled dataset were estimated using meta-analytical methods. Standard error estimation accounted for data clustering in survey designs. Stata 12 (STATA Corp, College Station, TX) was used for all analyses.

We also crudely estimated total pediatric fevers attending and tested at different sources of care in 2010 across studied countries to further contextualize findings in our discussion. This was done by applying proportions tested from our analysis to published estimates of total pediatric fevers updated to 2010 [46]. This crude analysis helps visualize the rough magnitude of tested and untested pediatric fevers at different sources of care in order to further inform discussion of results.

## Results

105,791 children under five years old were surveyed in 13 countries (Table 1). 27,916 (26.5%, 95% CI: 21.0%–32.0%) had reported fever in the two weeks prior to the survey interview, and 4,990 (16.9%, 95% CI: 11.8%–21.9%) were tested.

Table 2 indicates that 35.3% (95% CI: 26.1%–44.6%) of febrile children attending hospitals were tested compared to 26.0% (95% CI: 18.2%–33.9%) visiting non-hospital formal medical sources, and 16.5% (95% CI: 10.6%–22.3%) visiting CHWs. 22.8% (95% CI: 14.5%–31.1%) of febrile children in low-risk areas were tested compared to 20.0% (95% CI: 13.6%–26.4%) in moderate stable transmission areas, and 16.3% (95% CI: 10.8%–21.7%) in high transmission settings. 11.1% (95% CI: 9.2%–13.1%) of febrile children in malaria-free areas were reportedly tested.

**Table 2:** Characteristics of febrile children less than five years old reportedly tested in 13 countries.

		*n* febrile under-fives^a^	Percent febrile under-fives tested (95% CI)^b^	
***Total***		***27,916***	***16.9***	***(11.8–21.9)***
**Source of care** c	Hospital	5,279	35.3	(26.1–44.6)
	Non-hospital formal medical	9,938	26.0	(18.2–33.9)
	Community health worker	381	16.5	(10.6–22.3)
	Pharmacy	1,742	6.2	(3.4–9.0)
	Other	1,769	6.9	(4.3–9.4)
	No care sought	8,618	3.3	(2.3–4.3)
**Malaria endemicity** d	No transmission	1,023	11.1	(9.2–13.1)
	Unstable transmission	7	42.9	(15.8–75.0)
	Low stable transmission	2,797	22.8	(14.5–31.1)
	Moderate stable transmission	12,211	20.0	(13.6–26.4)
	High stable transmission	11,287	16.3	(10.8–21.7)
**Child's age (in months)**	0–5	2,174	12.7	(8.3–17.0)
	6–11	4,094	17.1	(11.6–22.7)
	12–23	7,191	18.1	(12.5–23.8)
	24–35	6,006	17.8	(12.3–23.2)
	36–47	4,782	16.0	(11.3–20.7)
	48–59	4,021	15.9	(10.7–21.1)
**Child's sex**	Male	14,297	17.0	(12.0–22.0)
	Female	13,620	16.7	(11.7–21.8)
**Maternal age (in years)**	15–24	8,798	17.7	(12.7–22.8)
	25–29	7,725	16.8	(11.1–22.5)
	30–34	5,237	16.4	(11.2–21.5)
	35–39	3,829	16.3	(11.3–21.3)
	40–49	2,331	16.3	(11.5–21.2)
**Maternal education**	No education	9,989	14.0	(9.8–18.2)
	Primary attendance	13,883	17.4	(13.1–21.7)
	Secondary or higher attendance	4,047	27.0	(18.6–35.5)
**Household wealth index**	Poorest	6,107	12.4	(8.6–16.3)
	Second	5,797	12.8	(9.1–16.5)
	Middle	5,838	14.6	(10.1–19.1)
	Fourth	5,609	18.5	(12.6–24.5)
	Least poor	4,574	27.6	(18.0–37.3)
**Number of household members**	0–4	7,239	18.1	(12.8–23.4)
	5–8	14,156	17.0	(12.1–21.9)
	9–12	4,241	16.3	(10.9–21.6)
	13 or more	2,280	14.6	(10.1–19.1)
**Residence**	Urban	5,651	27.4	(17.8–37.1)
	Rural	22,264	14.6	(10.4–18.7)

a Children less than five years old reportedly having fever in the 2 weeks prior to the interview.

b Febrile children less than five years old reportedly receiving a finger or heel stick for testing.

c Non-hospital formal medical refers to any formal medical source that is not a hospital or CHW. Other refers to traditional practitioners, shops, relatives/friends, or other non-specified locations.

d No transmission refer to non-endemic areas. Unstable transmission refers to areas of very low but non-zero malaria transmission. Stable transmission categories refer to low (*Pf*PR_2–10_<5%), moderate (*Pf*PR_2–10_ 5%–40%) and high (*Pf*PR_2–10_>40%).

### Main Covariates

Febrile children in high-risk areas were less often tested than those in low-risk areas (Table 3). Compared to low-risk areas, the odds of testing declined by 49% for febrile children in high-risk areas (OR: 0.51, 95% CI: 0.42–0.62), and by 54% (OR: 0.46, 95% CI: 0.34–0.63) in malaria-free areas. There was a non-significant difference in the odds of testing febrile children in moderate stable transmission areas when compared to low-risk areas (OR: 1.04, 95% CI: 0.86–1.25). Comparisons with unstable transmission areas are limited given few observations in these areas in our analysis.

**Table 3:** Effect of source of care, malaria endemicity and socioeconomic covariates on test uptake.

		AOR^a^	95% CI	p-value
**Source of care** b	Hospital	1.00		
	Non-hospital formal medical	0.62	0.56–0.69	<0.001
	Community health worker	0.31	0.23–0.43	<0.001
	Pharmacy	0.06	0.05–0.09	<0.001
	Other	0.10	0.08–0.13	<0.001
	No care sought	0.05	0.04–0.06	<0.001
**Malaria endemicity** c	No transmission	0.46	0.34–0.63	<0.001
	Unstable transmission	1.32	0.11–15.50	0.823
	Low stable transmission	1.00		
	Moderate stable transmission	1.04	0.86–1.25	0.697
	High stable transmission	0.51	0.42–0.62	<0.001
**Child's age (in months)**	0–5	0.72	0.59–0.87	0.001
	6–11	1.00		
	12–23	1.24	1.09–1.41	0.001
	24–35	1.27	1.11–1.45	<0.001
	36–47	1.10	0.95–1.26	0.203
	48–59	1.18	1.02–1.37	0.030
**Child's sex**	Male	1.00		
	Female	0.98	0.91–1.06	0.676
**Maternal age (in years)**	15–24	1.00		
	25–29	1.01	0.91–1.12	0.891
	30–34	1.06	0.94–1.20	0.336
	35–39	1.06	0.92–1.21	0.425
	40–49	0.99	0.83–1.17	0.890
**Maternal education**	No education attendance	1.00		
	Primary attendance	1.32	1.19–1.46	<0.001
	Secondary or higher attendance	1.33	1.16–1.54	<0.001
**Household wealth index**	Poorest	1.00		
	Second	0.99	0.87–1.13	0.850
	Middle	1.03	0.90–1.18	0.670
	Fourth	1.21	1.06–1.40	0.006
	Least poor	1.63	1.39–1.91	<0.001
**Number of household members**	0–4	1.00		
	5–8	0.95	0.86–1.05	0.307
	9–12	0.87	0.76–0.99	0.036
	13 or more	0.66	0.54–0.80	<0.001
**Residence**	Urban	1.00		
	Rural	0.71	0.62–0.82	<0.001

CI refers to confidence interval. AOR refers to adjusted odds ratio. COR refers to crude odds ratio.

a Mixed-effects logistic regression model in pooled dataset of 13 surveys, adjusted for data clustering and above covariates.

b COR (source of care): non-hospital = 0.56 (95% CI: 0.51–0.62); community health worker = 0.30 (95% CI: 0.21–0.41); pharmacy = 0.06 (95% CI: 0.05–0.08); other = 0.09 (95% CI: 0.07–0.12); no care sought = 0.04 (95% CI: 0.04–0.05). Non-hospital formal medical refers to any formal medical source that is not a hospital or CHW. Other refers to traditional practitioners, shops, relatives/friends, or other non-specified locations.

c COR (malaria endemicity): no transmission = 0.51 (95% CI: 0.38–0.70); unstable transmission = 5.67 (95% CI: 0.44–73.6); moderate stable transmission = 1.35 (95% CI: 1.12–1.63); high stable transmission = 0.67 (95% CI: 0.55–0.81). No risk areas refer to non-endemic areas. Unstable malaria transmission refers to areas of very low but non-zero transmission. Stable transmission categories refer to low (*Pf*PR_2–10_<5%), moderate (*Pf*PR_2–10_ 5%–40%) and high (*Pf*PR_2–10_>40%).

**Figure 2:**
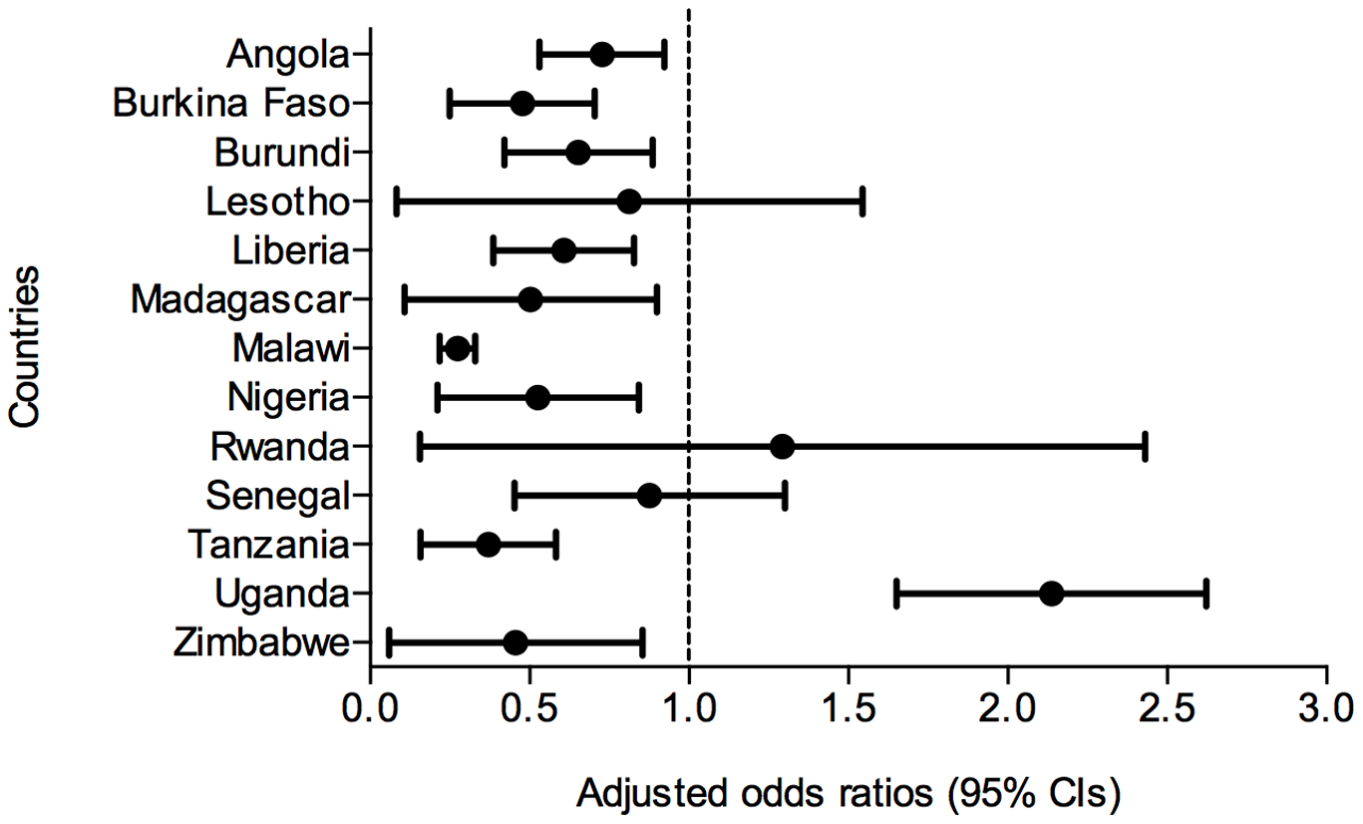
Forest plot of test uptake at non-hospital sources versus hospitals in each country. Figure legend: CI refers to confidence interval. Mixed-effects logistic regression models adjusted for data clustering and Table 3 covariates. AOR <1.0 indicates reduced odds of testing at non-hospital sources compared to hospitals.

**Figure 3:**
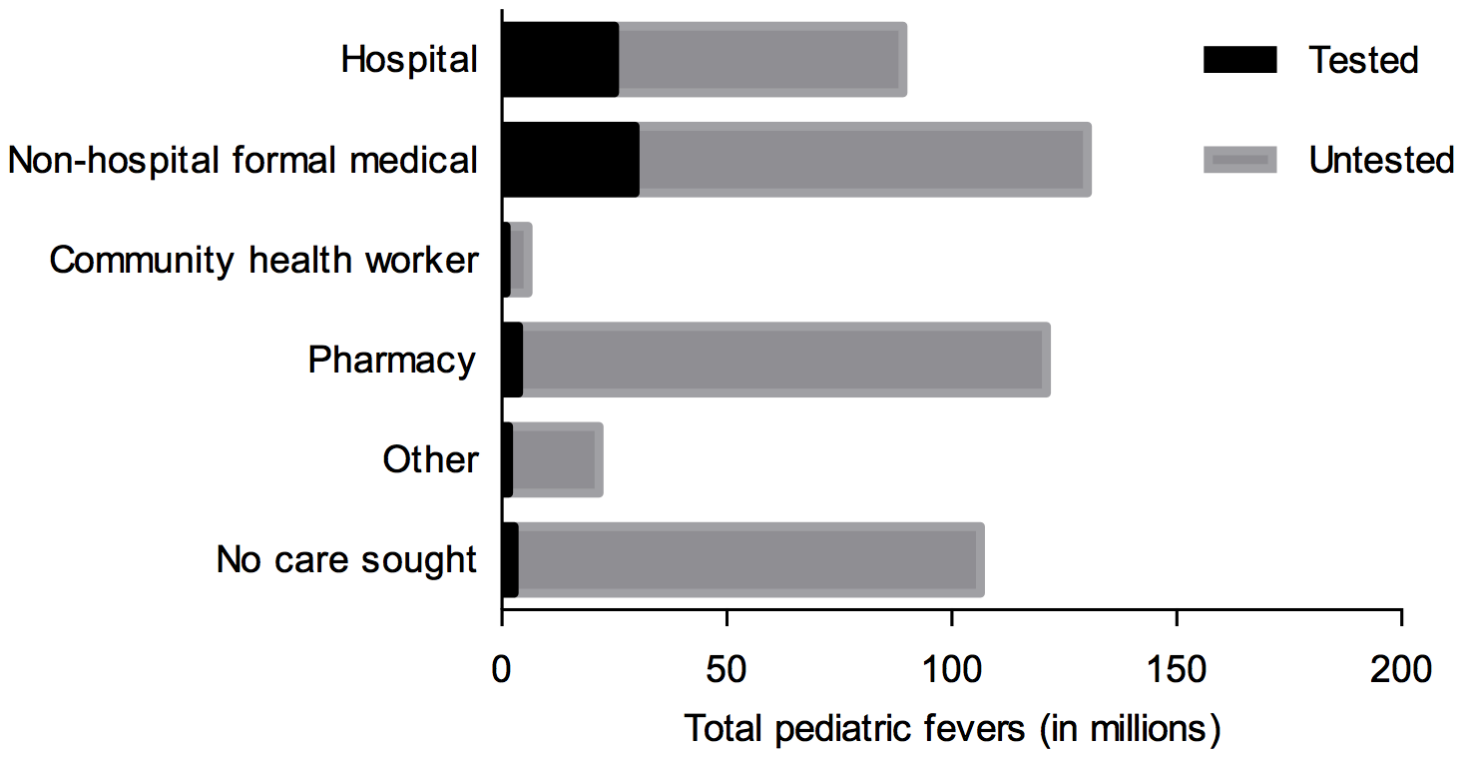
Estimated pediatric fevers attending and tested by source of care in 13 countries in 2010. Figure legend: All totals are given in ‘000 s.

Source of care was consistently and significantly associated with malaria test uptake after controlling for other covariates (Table 3). Compared to hospitals, the odds of testing febrile children decreased by 38% if attending non-hospital sources (OR: 0.62, 95% CI: 0.56–0.69), and by 69% (OR: 0.31, 95% CI: 0.23–0.43) if visiting CHWs. Nine countries had similar results (Figure 2 and Table S1). In Uganda, however, the odds of testing febrile children visiting non-hospital formal medical sources was 2.10 (95% CI: 1.67–2.64) times higher than if visiting hospitals. Figure 3 further illustrates the rough magnitude of tested and untested pediatric fevers at different sources of care in studied countries in 2010 to provide context to regression model results.

Crude analyses indicate a non-significant difference in test uptake among febrile children attending public (34.8%, 95% CI: 25.0%–44.5%) versus private (36.5%, 95% CI: 26.3%–46.8%) hospitals across studied countries (*p* = 0.315), and lower uptake at public (25.0%, 95% CI: 17.5%–32.5%) compared to private (30.8%, 95% CI: 16.5%–45.1%) non-hospital sources (*p* = 0.022).

### Other Covariates

Residence was significantly associated with test use in the adjusted analysis (Table 3). Compared to urban areas, the odds of testing febrile children decreased by 29% in rural settings (OR: 0.71, 95% CI: 0.62–0.82). For febrile children in least poor households, the odds of testing was 1.63 (95% CI: 1.39–1.91) times higher than for those in poorest households after adjusting for other covariates. Febrile children of mothers that attended primary or at least secondary education had 1.32 (95% CI: 1.19–1.46) and 1.33 (95% CI: 1.16–1.54) times higher odds of getting tested, respectively, than those having mothers with no education. Febrile children over 12 months were more often tested than infants aged 0–11 months. Compared to older infants (6–11 months), the odds of younger infants (0–5 months) getting tested declined by 28% in the adjusted analysis (OR: 0.72, 95% CI: 0.59–0.87). Maternal age and child’s sex were non-significant covariates.

### Stratification by Malaria Endemicity

**Figure 4:**
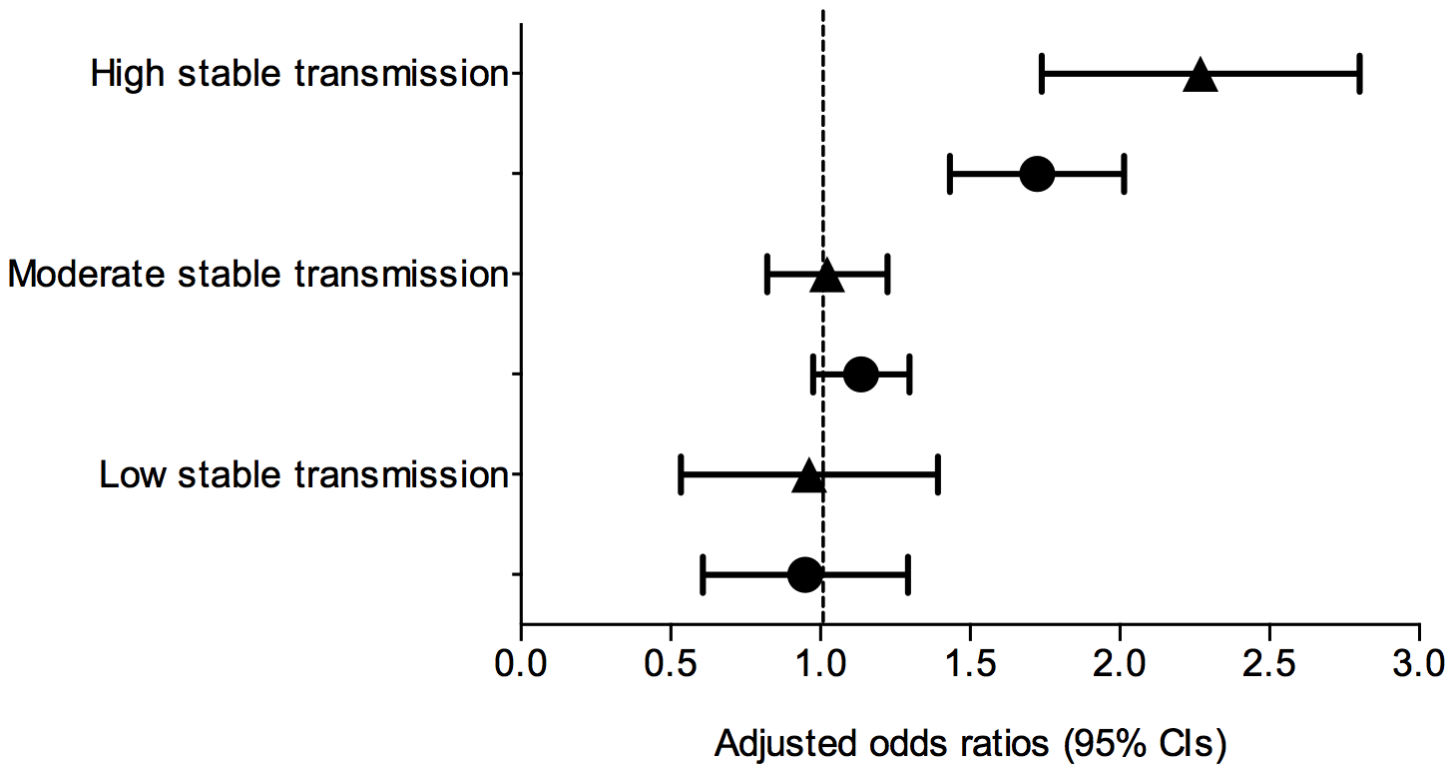
Effect of maternal education on test uptake in different malaria endemicities. Figure legend: ▴, Secondary or higher schooling versus no schooling; •, Primary schooling versus no schooling. Mixed-effects logistic regression model in pooled dataset of 13 surveys, adjusted for data clustering and Table 3 covariates. Stable transmission categories refer to low (*Pf*PR_2–10_<5%), moderate (*Pf*PR_2–10_ 5%–40%) and high (*Pf*PR_2–10_>40%).

There was evidence of an interaction between categorical variables maternal education and malaria endemicity (*p*-values ranged from 0.009 to 0.467 for stable transmission categories) when incorporated into the final model. To further explore this result, the final model was stratified by low (*Pf*PR_2–10_<5%), medium (*Pf*PR_2–10_ 5%–40%), and high (*Pf*PR_2–10_>40%) stable malaria transmission categories. In high-risk settings, the odds of testing febrile children with mothers who attended primary or at least secondary education was 1.71 (95% CI: 1.44–2.02) and 2.23 (95% CI: 1.76–2.82) times higher, respectively, than if the mother had no education after controlling for other covariates (Figure 4). This effect was negligible in moderate- and low-risk areas. The stratified model by urban and rural residence indicated no significant difference in maternal education’s effect on test use between contexts (data not shown).

## Discussion

Overall, diagnostic testing of pediatric fevers was low and inequitable in studied countries at the outset of new guidelines. Test uptake was lowest at locations where pediatric fevers are more often managed, particularly for poorest children, and in areas with the highest risk of malaria infection. Our findings also demonstrate an important socioeconomic dimension to malaria testing.

We found seeking care from lower levels or less formal sources greatly reduced the likelihood of testing, as occurs with other facility-based interventions [47]. This is most plausibly explained by lower test availability at these locations, including their near absence among CHWs and pharmacies [48]. It is also possible that patients attending hospitals are systematically different from those attending other sources in ways that influence testing (e.g. more severe illness).

Crude estimates of total pediatric fevers attending and tested at different sources of care further illustrates that diagnostic testing is not reaching sources of care bearing the disproportionate burden of fever cases. This is a particular challenge to improve equitable access to diagnostic testing in the future since these are the same sources where poor and marginalized families often seek care [32]. There is an urgent need to improve quantification of diagnostic need along these lines in order to inform mRDT forecasting and procurement as countries work toward universal test coverage.

Our results also indicate febrile children at the highest malaria risk are less often tested than those at lower risk. Some countries have prioritized mRDT to low-risk areas to increase diagnostic availability in these settings [49]. Reduced uptake in high-risk areas could also be due to entrenched presumptive treatment practices [50]. In locations where diagnostic tests commonly indicate malaria infection – or as malaria ‘suspicion’ rises – there may be less perceived value of testing over habitual presumptive treatment practices by caregivers and/or health workers. Similarly, less testing in malaria-free areas is likely due to lower malaria ‘suspicion’ in these settings. This finding merits further investigation given potential implications for mRDT scale-up in high-risk areas.

Different influences of maternal education on test use in low- and high-risk settings could further support this theory. Independent of other factors, our findings show febrile children of educated mothers in high-risk areas have twice the odds of getting tested than those with non-educated mothers, while this effect was negligible in low- and moderate-risk settings. Again, a perceived lesser value of testing in high-risk areas could exacerbate an ‘early adopter’ effect among better-educated mothers [51]. Poorly educated mothers, or those less open to new technologies or medical procedures, could be less inclined to change treatment habits in areas where testing commonly provides the same result as presumptive practices, particularly if time or cost is associated with testing. Health workers, too, could less often test children in high-risk settings without caregiver demand, which favors educated mothers. In low-risk areas where a malaria diagnosis is less clear, a wider range of caregivers and/or health workers may be more inclined toward testing, potentially coupled with higher test availability depending on country implementation strategies.

Results show infants are less often tested than older children, and younger infants (0–5 months) are less often tested than older ones (six to 11 months). This finding has not been previously reported to our knowledge. Mean age of malaria onset is about six months [52]. Health workers may therefore not suspect malaria in young infants and test less often. Alternatively, fever in infants is often a clinical ‘red flag’ given higher mortality rates in this age group. This could cause backsliding to habitual presumptive treatment practices. This result merits further investigation since malaria infection is still possible in young infants. In fact, testing could arguably be more informative for this group since diagnosis is less clear, and differential diagnosis of childhood illnesses with overlapping symptoms (e.g. malaria and pneumonia) is important [53].

Our study further demonstrates a socioeconomic dimension to malaria testing. Independent of other factors, febrile children in poorest households are less often tested, as are those with poorly educated mothers. Rural settings are associated with less testing, as are large households in the adjusted analysis. This is likely due to lower test availability where marginalized families often seek care. Integrated community case management is a promising approach to improve equitable access to testing and care [54]. Studies show that CHWs can appropriately use rapid diagnostic tests to manage pediatric fevers [55].

These results should be viewed in light of some data limitations. First, findings indicate differences in test uptake across population groups but do not explain reasons for observed practices. Second, surveys only measure whether blood was taken for testing, and as such, do not differentiate between microscopy and mRDT. Higher coverage at certain locations, such as hospitals, may be due to long-standing microscopy availability rather than targeted mRDT roll out. Moreover, testing practices could differ for microscopy and mRDT, particularly since mRDT requires less training and time to use effectively [56]–[58]. Data in this analysis may largely reflect testing by microscopy given our early assessment with countries at different stages of mRDT implementation. Future analyses based on more recent data, once available, could potentially provide a different result if a greater proportion of testing is conducted using mRDT rather than microscopy. Third, data indicate test use but not appropriate treatment based on test results. Fourth, surveys do not measure facility or clinician factors that may greatly influence uptake.

Finally, a recent validation study found caregiver recall of testing was not highly sensitive (61.9%) but had reasonable specificity (90.0%) when compared to direct facility observation of malaria diagnostic test receipt [59]. The authors found no significant differences in recall across examined caregiver characteristics. Other studies have shown poor caregiver recall of child morbidities or previous health events, particularly among poor, rural or less educated mothers [60], [61]. Findings could overestimate differences in test uptake between these groups.

## Conclusion

Based on 105,791 children under age five years surveyed in 13 countries in 2009–2011/12, our findings demonstrate low and inequitable testing of pediatric fevers as countries start to implement new guidelines. Malaria diagnostic testing has become increasingly important in the context of malaria control and elimination to improve surveillance, rational drug use and appropriate fever management [62]. Countries are now working toward universal test coverage of all suspected malaria cases in line with revised international guidelines. This paper represents an early assessment against which to measure future progress in diagnostic scale-up, and highlights inequities in testing that need to be addressed going forward. Research is urgently needed to better understand reasons for reduced testing among the youngest children and in high-risk settings, which could plausibly be due to a perceived lesser value of testing for these populations. This analysis should be repeated in the near-term as mRDT implementation matures, and additional data become available for the years 2012–2014. Similar analyses are also needed to examine testing practices for older children and adults. Current findings could inform continued mRDT implementation in order to improve access to and equity in point-of-care diagnostics use for pediatric fevers.

## Supporting Information

Table S1 **National results for the effect of source of care, malaria endemicity and socioeconomic covariates on test uptake.** Table legend: CI refers to confidence interval. AOR refers to adjusted odds ratio. Mixed-effects logistic regression models in individual country datasets, adjusted for data clustering and all listed covariates.
(DOCX)

Checklist S1 **PRISMA checklist.**
(DOC)
