# Supplementary material for: Diagnostic Testing of Pediatric Fevers: Meta-Analysis of 13 National Surveys Assessing Influences of Malaria Endemicity and Source of Care on Test Uptake for Febrile Children under Five Years
Source: PLoS One. 2014 Apr 18;9(4):e95483. doi: 10.1371/journal.pone.0095483 (PMC3991688; doi:10.1371/journal.pone.0095483)
Supplement: Table S1 — National results for the effect of source of care, malaria endemicity and socioeconomic covariates on test uptake. Table legend: CI refers to confidence interval. AOR refers to adjusted odds ratio. Mixed-effects logistic regression models in individual country datasets, adjusted for data clustering and all listed covariates. (DOCX) [file pone.0095483.s001.docx]

| **Angola**  **Malaria Indicator Survey 2011** |  | **AOR** | **95% CI** | | **pvalue** |
| --- | --- | --- | --- | --- | --- |
| **Level of care** | Hospital | 1.00 |  |  |  |
|  | Non-hospital formal medical | 0.71 | 0.54 | 0.93 | 0.013 |
|  | Pharmacy or other | 0.01 | 0.00 | 0.06 | <0.001 |
|  | No care sought | 0.02 | 0.01 | 0.04 | <0.001 |
| **Malaria endemicity** | No transmission | 0.36 | 0.04 | 3.33 | 0.369 |
|  | Low stable transmission | 1.00 |  |  |  |
|  | Moderate stable transmission | 0.78 | 0.24 | 2.50 | 0.672 |
|  | High stable transmission | 1.07 | 0.31 | 3.67 | 0.913 |
| **Child's age (in months)** | 0 – 5 | 0.88 | 0.52 | 1.48 | 0.636 |
|  | 6 – 11 | 1.00 |  |  |  |
|  | 12 - 23 | 1.44 | 0.98 | 2.11 | 0.062 |
|  | 24 - 35 | 1.38 | 0.93 | 2.05 | 0.111 |
|  | 36 - 47 | 1.67 | 1.07 | 2.60 | 0.024 |
|  | 48 - 59 | 1.56 | 0.99 | 2.46 | 0.057 |
| **Child's sex** | Male | 1.00 |  |  |  |
|  | Female | 1.01 | 0.80 | 1.28 | 0.909 |
| **Maternal age (in years)** | 15 -24 | 1.00 |  |  |  |
|  | 25 - 29 | 1.13 | 0.83 | 1.55 | 0.431 |
|  | 30 - 34 | 1.54 | 1.07 | 2.21 | 0.019 |
|  | 35 - 39 | 1.17 | 0.78 | 1.76 | 0.447 |
|  | 40 - 49 | 0.65 | 0.36 | 1.16 | 0.145 |
| **Maternal education** | None | 1.00 |  |  |  |
|  | Primary attendance | 1.43 | 1.04 | 1.97 | 0.027 |
|  | At least secondary attendance | 1.49 | 0.92 | 2.41 | 0.108 |
| **Household wealth index** | Poorest | 1.00 |  |  |  |
|  | Second | 1.36 | 0.79 | 2.36 | 0.267 |
|  | Middle | 1.39 | 0.81 | 2.38 | 0.231 |
|  | Fourth | 1.89 | 1.10 | 3.25 | 0.021 |
|  | Least poor | 1.93 | 1.10 | 3.38 | 0.022 |
| **Total household members** | 1-4 members | 1.00 |  |  |  |
|  | 5-8 members | 0.90 | 0.67 | 1.20 | 0.465 |
|  | 9-12 members | 0.91 | 0.61 | 1.36 | 0.642 |
|  | 13 or more members | 0.94 | 0.39 | 2.23 | 0.882 |
| **Residence** | Urban | 1.00 |  |  |  |
|  | Rural | 0.30 | 0.21 | 0.44 | <0.001 |

Table legend:

AOR based on mixed-effects logistic regression models adjusted for data clustering and confounding covariates. PSUs included in model as random effects. All listed covariates included as fixed categorical effects.

**Burkina Faso**

**Demographic and Health Survey 2010-2011**

|  |  | **AOR** | **95% CI** | | **pvalue** |  |
| --- | --- | --- | --- | --- | --- | --- |
| **Level of care** | Hospital | 1.00 |  |  |  |  |
|  | Non-hospital formal medical | 0.44 | 0.27 | 0.72 | 0.001 |  |
|  | CHW | 0.63 | 0.12 | 3.38 | 0.590 |  |
|  | Pharmacy | 0.28 | 0.07 | 1.05 | 0.059 |  |
|  | Other | 0.06 | 0.02 | 0.22 | <0.001 |  |
|  | No care sought | 0.05 | 0.02 | 0.12 | <0.001 |  |
| **Malaria endemicity** | High stable transmission | 1.00 |  |  |  |  |
| **Child's age (in months)** | 0 – 5 | 0.94 | 0.34 | 2.59 | 0.897 |  |
|  | 6 - 11 | 1.00 |  |  |  |  |
|  | 12 - 23 | 1.32 | 0.72 | 2.43 | 0.371 |  |
|  | 24 - 35 | 1.47 | 0.78 | 2.78 | 0.233 |  |
|  | 36 - 47 | 1.19 | 0.58 | 2.43 | 0.634 |  |
|  | 48 - 59 | 0.74 | 0.31 | 1.74 | 0.487 |  |
| **Child's sex** | Male | 1.00 |  |  |  |  |
|  | Female | 1.23 | 0.84 | 1.80 | 0.281 |  |
| **Maternal age (in years)** | 15 -24 | 1.00 |  |  |  |  |
|  | 25 - 29 | 0.49 | 0.27 | 0.89 | 0.020 |  |
|  | 30 - 34 | 1.55 | 0.90 | 2.67 | 0.118 |  |
|  | 35 - 39 | 1.09 | 0.56 | 2.12 | 0.805 |  |
|  | 40 - 49 | 1.28 | 0.59 | 2.77 | 0.534 |  |
| **Maternal education** | None | 1.00 |  |  |  |  |
|  | Primary attendance | 1.90 | 1.14 | 3.15 | 0.013 |  |
|  | At least secondary attendance | 1.73 | 0.82 | 3.64 | 0.152 |  |
| **Household wealth index** | Poorest | 1.00 |  |  |  |  |
|  | Second | 0.95 | 0.48 | 1.89 | 0.879 |  |
|  | Middle | 0.68 | 0.34 | 1.36 | 0.275 |  |
|  | Fourth | 0.77 | 0.39 | 1.54 | 0.466 |  |
|  | Least poor | 0.79 | 0.35 | 1.82 | 0.588 |  |
| **Total household members** | 1-4 members | 1.00 |  |  |  |  |
|  | 5-8 members | 0.83 | 0.50 | 1.37 | 0.460 |  |
|  | 9-12 members | 0.56 | 0.29 | 1.09 | 0.086 |  |
|  | 13 or more members | 0.60 | 0.28 | 1.31 | 0.204 |  |
| **Residence** | Urban | 1.00 |  |  |  |  |
|  | Rural | 1.01 | 0.55 | 1.89 | 0.963 |  |
| Table legend:  AOR based on mixed-effects logistic regression models adjusted for data clustering and confounding covariates. PSUs included in model as random effects. All listed covariates included as fixed categorical effects. | | | | | | |

**Burundi**

**Demographic and Health Survey 2010-2011**

|  |  | **AOR** | **95% CI** | | **pvalue** |
| --- | --- | --- | --- | --- | --- |
| **Level of care** | Hospital | 1.00 |  |  |  |
|  | Non-hospital formal medical | 0.62 | 0.44 | 0.90 | 0.011 |
|  | CHW | 0.64 | 0.23 | 1.76 | 0.386 |
|  | Pharmacy | 0.10 | 0.04 | 0.27 | <0.001 |
|  | Other | 0.26 | 0.10 | 0.68 | 0.006 |
|  | No care sought | 0.07 | 0.04 | 0.10 | <0.001 |
| **Malaria endemicity** | No transmission | 0.21 | 0.08 | 0.55 | 0.001 |
|  | Low stable transmission | 1.00 |  |  |  |
|  | Moderate stable transmission | 0.92 | 0.53 | 1.59 | 0.765 |
|  | High stable transmission | 3.11 | 0.54 | 17.75 | 0.202 |
| **Child's age (in months)** | 0 - 5 | 0.49 | 0.27 | 0.88 | 0.018 |
|  | 6 - 11 | 1.00 |  |  |  |
|  | 12 - 23 | 1.91 | 1.29 | 2.82 | 0.001 |
|  | 24 - 35 | 1.54 | 1.02 | 2.33 | 0.039 |
|  | 36 - 47 | 1.51 | 0.98 | 2.31 | 0.059 |
|  | 48 - 59 | 1.74 | 1.09 | 2.78 | 0.021 |
| **Child's sex** | Male | 1.00 |  |  |  |
|  | Female | 1.11 | 0.87 | 1.41 | 0.409 |
| **Maternal age (in years)** | 15 -24 | 1.00 |  |  |  |
|  | 25 - 29 | 0.85 | 0.61 | 1.21 | 0.371 |
|  | 30 - 34 | 0.87 | 0.58 | 1.29 | 0.478 |
|  | 35 - 39 | 0.81 | 0.53 | 1.25 | 0.339 |
|  | 40 - 49 | 0.88 | 0.53 | 1.46 | 0.615 |
| **Maternal education** | None | 1.00 |  |  |  |
|  | Primary attendance | 1.23 | 0.94 | 1.61 | 0.131 |
|  | At least secondary attendance | 1.72 | 1.02 | 2.89 | 0.042 |
| **Household wealth index** | Poorest | 1.00 |  |  |  |
|  | Second | 1.36 | 0.92 | 2.01 | 0.124 |
|  | Middle | 1.75 | 1.17 | 2.62 | 0.006 |
|  | Fourth | 1.85 | 1.23 | 2.76 | 0.003 |
|  | Least poor | 1.46 | 0.91 | 2.35 | 0.120 |
| **Total household members** | 1-4 members | 1.00 |  |  |  |
|  | 5-8 members | 0.95 | 0.70 | 1.30 | 0.767 |
|  | 9-12 members | 0.89 | 0.55 | 1.46 | 0.653 |
|  | 13 or more members | 0.69 | 0.20 | 2.35 | 0.552 |
| **Residence** | Urban | 1.00 |  |  |  |
|  | Rural | 0.46 | 0.28 | 0.76 | 0.003 |

Table legend:

AOR based on mixed-effects logistic regression models adjusted for data clustering and confounding covariates. PSUs included in model as random effects. All listed covariates included as fixed categorical effects.

**Lesotho**

**Demographic and Health Survey 2009-2010**

|  |  | **AOR** | **95% CI** | | **pvalue** |
| --- | --- | --- | --- | --- | --- |
| **Level of care** | Hospital | 1.00 |  |  |  |
|  | Non-hospital formal medical | 0.59 | 0.22 | 1.63 | 0.312 |
|  | CHW | 1.19 | 0.26 | 5.52 | 0.825 |
|  | Pharmacy or other | 0.59 | 0.14 | 2.51 | 0.472 |
|  | No care sought | 0.32 | 0.11 | 0.94 | 0.038 |
| **Malaria endemicity** | No transmission | 1.00 |  |  |  |
| **Child's age (in months)** | 0 – 5 | 0.39 | 0.10 | 1.59 | 0.190 |
|  | 6 - 11 | 1.00 |  |  |  |
|  | 12 - 23 | 0.52 | 0.20 | 1.36 | 0.183 |
|  | 24 - 35 | 0.62 | 0.22 | 1.78 | 0.374 |
|  | 36 - 47 | 0.37 | 0.11 | 1.27 | 0.115 |
|  | 48 - 59 | 0.64 | 0.19 | 2.22 | 0.485 |
| **Child's sex** | Male | 1.00 |  |  |  |
|  | Female | 1.01 | 0.53 | 1.95 | 0.974 |
| **Maternal age (in years)** | 15 -24 | 1.00 |  |  |  |
|  | 25 - 29 | 1.33 | 0.54 | 3.28 | 0.535 |
|  | 30 - 34 | 1.53 | 0.59 | 4.00 | 0.385 |
|  | 35 - 39 | 0.88 | 0.26 | 3.01 | 0.838 |
|  | 40 - 49 | 2.48 | 0.71 | 8.65 | 0.155 |
| **Maternal education** | None | 1.00 |  |  |  |
|  | Primary attendance | 0.42 | 0.06 | 2.95 | 0.386 |
|  | At least secondary attendance | 0.49 | 0.07 | 3.74 | 0.495 |
| **Household wealth index** | Poorest | 1.00 |  |  |  |
|  | Second | 1.15 | 0.45 | 2.91 | 0.773 |
|  | Middle | 1.04 | 0.38 | 2.85 | 0.933 |
|  | Fourth | 1.29 | 0.40 | 4.11 | 0.670 |
|  | Least poor | 1.67 | 0.38 | 7.44 | 0.499 |
| **Total household members** | 1-4 members | 1.00 |  |  |  |
|  | 5-8 members | 1.66 | 0.68 | 4.09 | 0.267 |
|  | 9-12 members | 1.01 | 0.30 | 3.36 | 0.989 |
|  | 13 or more members | 2.58 | 0.45 | 14.62 | 0.285 |
| **Residence** | Urban | 1.00 |  |  |  |
|  | Rural | 1.79 | 0.45 | 7.14 | 0.411 |

Table legend:

AOR based on mixed-effects logistic regression models adjusted for data clustering and confounding covariates. PSUs included in model as random effects. All listed covariates included as fixed categorical effects.

**Liberia**

**Malaria Indicator Survey 2011**

|  |  | **AOR** | **95% CI** | | **pvalue** |
| --- | --- | --- | --- | --- | --- |
| **Level of care** | Hospital | 1.00 |  |  |  |
|  | Non-hospital formal medical | 0.58 | 0.40 | 0.84 | 0.004 |
|  | CHW or pharmacy or other | 0.05 | 0.03 | 0.09 | <0.001 |
|  | No care sought | 0.01 | 0.01 | 0.03 | <0.001 |
| **Malaria endemicity** | Moderate stable transmission | 1.00 |  |  |  |
|  | High stable transmission | 1.05 | 0.68 | 1.63 | 0.810 |
| **Child's age (in months)** | 0 - 5 | 0.59 | 0.32 | 1.10 | 0.097 |
|  | 6 - 11 | 1.00 |  |  |  |
|  | 12 - 23 | 1.24 | 0.76 | 2.01 | 0.394 |
|  | 24 - 35 | 1.43 | 0.86 | 2.36 | 0.166 |
|  | 36 - 47 | 1.46 | 0.87 | 2.43 | 0.150 |
|  | 48 - 59 | 1.67 | 0.99 | 2.82 | 0.054 |
| **Child's sex** | Male | 1.00 |  |  |  |
|  | Female | 1.18 | 0.90 | 1.55 | 0.231 |
| **Maternal age (in years)** | 15 -24 | 1.00 |  |  |  |
|  | 25 - 29 | 0.86 | 0.60 | 1.25 | 0.440 |
|  | 30 - 34 | 1.03 | 0.67 | 1.58 | 0.906 |
|  | 35 - 39 | 0.88 | 0.56 | 1.40 | 0.597 |
|  | 40 - 49 | 0.93 | 0.54 | 1.58 | 0.778 |
| **Maternal education** | None | 1.00 |  |  |  |
|  | Primary attendance | 1.24 | 0.89 | 1.73 | 0.212 |
|  | At least secondary attendance | 1.14 | 0.77 | 1.70 | 0.518 |
| **Household wealth index** | Poorest | 1.00 |  |  |  |
|  | Second | 0.78 | 0.51 | 1.18 | 0.233 |
|  | Middle | 0.94 | 0.57 | 1.56 | 0.816 |
|  | Fourth | 0.98 | 0.53 | 1.79 | 0.936 |
|  | Least poor | 1.37 | 0.69 | 2.74 | 0.370 |
| **Total household members** | 1-4 members | 1.00 |  |  |  |
|  | 5-8 members | 1.11 | 0.79 | 1.55 | 0.559 |
|  | 9-12 members | 0.83 | 0.51 | 1.33 | 0.434 |
|  | 13 or more members | 0.63 | 0.32 | 1.22 | 0.168 |
| **Residence** | Urban | 1.00 |  |  |  |
|  | Rural | 0.78 | 0.47 | 1.29 | 0.327 |

Table legend:

AOR based on mixed-effects logistic regression models adjusted for data clustering and confounding covariates. PSUs included in model as random effects. All listed covariates included as fixed categorical effects.

**Madagascar**

**Malaria Indicator Survey 2011**

|  |  | **AOR** | **95% CI** | | **pvalue** |
| --- | --- | --- | --- | --- | --- |
| **Level of care** | Hospital | 1.00 |  |  |  |
|  | Non-hospital formal medical | 0.40 | 0.17 | 0.94 | 0.035 |
|  | CHW | 0.45 | 0.07 | 2.99 | 0.408 |
|  | Pharmacy or other | 0.03 | 0.00 | 0.25 | 0.001 |
|  | No care sought | 0.04 | 0.01 | 0.11 | <0.001 |
| **Malaria endemicity** | Moderate stable transmission | 1.00 |  |  |  |
|  | High stable transmission | 2.89 | 1.28 | 6.55 | 0.011 |
| **Child's age (in months)** | 0 - 5 | 0.86 | 0.18 | 4.15 | 0.851 |
|  | 6 - 11 | 1.00 |  |  |  |
|  | 12 - 23 | 0.50 | 0.14 | 1.75 | 0.279 |
|  | 24 - 35 | 1.78 | 0.59 | 5.32 | 0.303 |
|  | 36 - 47 | 1.41 | 0.44 | 4.55 | 0.563 |
|  | 48 - 59 | 0.89 | 0.22 | 3.65 | 0.874 |
| **Child's sex** | Male | 1.00 |  |  |  |
|  | Female | 0.58 | 0.28 | 1.20 | 0.145 |
| **Maternal age (in years)** | 15 -24 | 1.00 |  |  |  |
|  | 25 - 29 | 0.31 | 0.10 | 0.96 | 0.043 |
|  | 30 - 34 | 0.47 | 0.16 | 1.38 | 0.167 |
|  | 35 - 39 | 1.28 | 0.46 | 3.58 | 0.633 |
|  | 40 - 49 | 0.56 | 0.15 | 2.11 | 0.394 |
| **Maternal education** | None | 1.00 |  |  |  |
|  | Primary attendance | 1.72 | 0.68 | 4.39 | 0.255 |
|  | At least secondary attendance | 1.25 | 0.36 | 4.33 | 0.722 |
| **Household wealth index** | Poorest | 1.00 |  |  |  |
|  | Second | 0.84 | 0.27 | 2.59 | 0.761 |
|  | Middle | 0.81 | 0.25 | 2.62 | 0.725 |
|  | Fourth | 1.38 | 0.44 | 4.33 | 0.583 |
|  | Least poor | 0.26 | 0.05 | 1.25 | 0.092 |
| **Total household members** | 1-4 members | 1.00 |  |  |  |
|  | 5-8 members | 1.07 | 0.46 | 2.49 | 0.869 |
|  | 9-12 members | 1.13 | 0.36 | 3.53 | 0.828 |
|  | 13 or more members | 1.07 | 0.90 | 12.58 | 0.959 |
| **Residence** | Urban | 1.00 |  |  |  |
|  | Rural | 0.61 | 0.20 | 1.86 | 0.382 |

Table legend:

AOR based on mixed-effects logistic regression models adjusted for data clustering and confounding covariates. PSUs included in model as random effects. All listed covariates included as fixed categorical effects.

**Malawi**

**Demographic and Health Survey 2010**

|  |  | **AOR** | **95% CI** | | **pvalue** |
| --- | --- | --- | --- | --- | --- |
| **Level of care** | Hospital | 1.00 |  |  |  |
|  | Non-hospital formal medical | 0.27 | 0.22 | 0.33 | <0.001 |
|  | Pharmacy or other | 0.10 | 0.07 | 0.15 | <0.001 |
|  | No care sought | 0.07 | 0.05 | 0.09 | <0.001 |
| **Malaria endemicity** | Moderate stable transmission | 1.00 |  |  |  |
|  | High stable transmission | 1.07 | 0.86 | 1.32 | 0.550 |
| **Child's age (in months)** | 0 - 5 | 0.86 | 0.58 | 1.26 | 0.435 |
|  | 6 - 11 | 1.00 |  |  |  |
|  | 12 - 23 | 0.93 | 0.72 | 1.20 | 0.563 |
|  | 24 - 35 | 1.10 | 0.85 | 1.43 | 0.466 |
|  | 36 - 47 | 0.94 | 0.71 | 1.25 | 0.666 |
|  | 48 - 59 | 0.84 | 0.63 | 1.13 | 0.254 |
| **Child's sex** | Male | 1.00 |  |  |  |
|  | Female | 0.92 | 0.78 | 1.08 | 0.293 |
| **Maternal age (in years)** | 15 -24 | 1.00 |  |  |  |
|  | 25 - 29 | 1.12 | 0.91 | 1.38 | 0.274 |
|  | 30 - 34 | 0.88 | 0.68 | 1.13 | 0.307 |
|  | 35 - 39 | 1.03 | 0.77 | 1.38 | 0.848 |
|  | 40 - 49 | 1.19 | 0.83 | 1.69 | 0.343 |
| **Maternal education** | None | 1.00 |  |  |  |
|  | Primary attendance | 0.92 | 0.72 | 1.17 | 0.498 |
|  | At least secondary attendance | 1.21 | 0.87 | 1.67 | 0.257 |
| **Household wealth index** | Poorest | 1.00 |  |  |  |
|  | Second | 1.09 | 0.85 | 1.40 | 0.495 |
|  | Middle | 1.13 | 0.88 | 1.43 | 0.339 |
|  | Fourth | 1.14 | 0.87 | 1.48 | 0.339 |
|  | Least poor | 1.84 | 1.34 | 2.52 | <0.001 |
| **Total household members** | 1-4 members | 1.00 |  |  |  |
|  | 5-8 members | 1.05 | 0.86 | 1.28 | 0.625 |
|  | 9-12 members | 0.95 | 0.70 | 1.29 | 0.739 |
|  | 13 or more members | 1.16 | 0.58 | 2.33 | 0.677 |
| **Residence** | Urban | 1.00 |  |  |  |
|  | Rural | 0.82 | 0.58 | 1.15 | 0.256 |

Table legend:

AOR based on mixed-effects logistic regression models adjusted for data clustering and confounding covariates. PSUs included in model as random effects. All listed covariates included as fixed categorical effects.

**Nigeria**

**Malaria Indicator Survey 2010**

|  |  | **AOR** | **95% CI** | | **pvalue** |
| --- | --- | --- | --- | --- | --- |
| **Level of care** | Hospital | 1.00 |  |  |  |
|  | Non-hospital formal medical | 0.46 | 0.25 | 0.87 | 0.017 |
|  | CHW | 0.55 | 0.13 | 2.42 | 0.430 |
|  | Pharmacy | 0.10 | 0.05 | 0.20 | <0.001 |
|  | Other | 0.19 | 0.06 | 0.63 | 0.007 |
|  | No care sought | 0.04 | 0.01 | 0.17 | <0.001 |
| **Malaria endemicity** | Moderate stable transmission | 1.00 |  |  |  |
|  | High stable transmission | 1.55 | 0.78 | 3.06 | 0.208 |
| **Child's age (in months)** | 0 - 5 | 1.70 | 0.51 | 5.65 | 0.386 |
|  | 6 - 11 | 1.00 |  |  |  |
|  | 12 - 23 | 1.81 | 0.80 | 4.10 | 0.156 |
|  | 24 - 35 | 1.14 | 0.47 | 2.79 | 0.767 |
|  | 36 - 47 | 1.62 | 0.69 | 3.80 | 0.273 |
|  | 48 - 59 | 0.91 | 0.35 | 2.36 | 0.840 |
| **Child's sex** | Male | 1.00 |  |  |  |
|  | Female | 1.14 | 0.72 | 1.81 | 0.574 |
| **Maternal age (in years)** | 15 -24 | 1.00 |  |  |  |
|  | 25 - 29 | 0.79 | 0.42 | 1.48 | 0.457 |
|  | 30 - 34 | 0.97 | 0.48 | 1.96 | 0.930 |
|  | 35 - 39 | 1.43 | 0.64 | 3.19 | 0.383 |
|  | 40 - 49 | 1.18 | 0.48 | 2.89 | 0.725 |
| **Maternal education** | None | 1.00 |  |  |  |
|  | Primary attendance | 1.24 | 0.59 | 2.58 | 0.573 |
|  | At least secondary attendance | 1.67 | 0.81 | 3.47 | 0.168 |
| **Household wealth index** | Poorest | 1.00 |  |  |  |
|  | Second | 0.61 | 0.24 | 1.54 | 0.296 |
|  | Middle | 0.82 | 0.34 | 1.94 | 0.645 |
|  | Fourth | 1.10 | 0.43 | 2.83 | 0.838 |
|  | Least poor | 1.43 | 0.49 | 4.18 | 0.519 |
| **Total household members** | 1-4 members | 1.00 |  |  |  |
|  | 5-8 members | 0.72 | 0.41 | 1.25 | 0.240 |
|  | 9-12 members | 0.38 | 0.16 | 0.88 | 0.023 |
|  | 13 or more members | 0.48 | 0.16 | 1.50 | 0.209 |
| **Residence** | Urban | 1.00 |  |  |  |
|  | Rural | 1.52 | 0.77 | 2.98 | 0.225 |

Table legend:

AOR based on mixed-effects logistic regression models adjusted for data clustering and confounding covariates. PSUs included in model as random effects. All listed covariates included as fixed categorical effects.

**Rwanda**

**Demographic and Health Survey 2010-2011**

|  |  | **AOR** | **95% CI** | | **pvalue** |
| --- | --- | --- | --- | --- | --- |
| **Level of care** | Hospital | 1.00 |  |  |  |
|  | Non-hospital formal medical | 0.96 | 0.36 | 2.56 | 0.938 |
|  | CHW | 0.11 | 0.04 | 0.32 | <0.001 |
|  | Pharmacy | 0.16 | 0.05 | 0.50 | 0.002 |
|  | Other | 0.07 | 0.02 | 0.26 | <0.001 |
|  | No care sought | 0.02 | 0.01 | 0.06 | <0.001 |
| **Malaria endemicity** | No transmission | 0.74 | 0.45 | 1.22 | 0.238 |
|  | Low stable transmission | 1.00 |  |  |  |
|  | Moderate stable transmission | 1.98 | 1.28 | 3.06 | 0.002 |
| **Child's age (in months)** | 0 - 5 | 0.44 | 0.17 | 1.09 | 0.077 |
|  | 6 - 11 | 1.00 |  |  |  |
|  | 12 - 23 | 1.26 | 0.74 | 2.13 | 0.395 |
|  | 24 - 35 | 1.01 | 0.58 | 1.77 | 0.963 |
|  | 36 - 47 | 0.67 | 0.36 | 1.25 | 0.211 |
|  | 48 - 59 | 0.83 | 0.43 | 1.61 | 0.587 |
| **Child's sex** | Male | 1.00 |  |  |  |
|  | Female | 0.79 | 0.56 | 1.12 | 0.192 |
| **Maternal age (in years)** | 15 -24 | 1.00 |  |  |  |
|  | 25 - 29 | 1.14 | 0.71 | 1.84 | 0.591 |
|  | 30 - 34 | 1.33 | 0.78 | 2.27 | 0.288 |
|  | 35 - 39 | 1.63 | 0.86 | 3.08 | 0.133 |
|  | 40 - 49 | 1.39 | 0.67 | 2.88 | 0.381 |
| **Maternal education** | None | 1.00 |  |  |  |
|  | Primary attendance | 1.07 | 0.64 | 1.81 | 0.786 |
|  | At least secondary attendance | 2.01 | 0.96 | 4.22 | 0.065 |
| **Household wealth index** | Poorest | 1.00 |  |  |  |
|  | Second | 0.87 | 0.50 | 1.52 | 0.628 |
|  | Middle | 0.92 | 0.53 | 1.61 | 0.771 |
|  | Fourth | 1.46 | 0.83 | 2.57 | 0.194 |
|  | Least poor | 2.23 | 1.21 | 4.11 | 0.010 |
| **Total household members** | 1-4 members | 1.00 |  |  |  |
|  | 5-8 members | 1.04 | 0.70 | 1.54 | 0.861 |
|  | 9-12 members | 0.83 | 0.39 | 1.74 | 0.621 |
|  | 13 or more members | 2.98 | 0.18 | 50.07 | 0.448 |
| **Residence** | Urban | 1.00 |  |  |  |
|  | Rural | 0.62 | 0.35 | 1.08 | 0.092 |

Table legend:

AOR based on mixed-effects logistic regression models adjusted for data clustering and confounding covariates. PSUs included in model as random effects. All listed covariates included as fixed categorical effects.

**Senegal**

**Demographic and Health Survey 2010-2011**

|  |  | **AOR** | **95% CI** | | **pvalue** |
| --- | --- | --- | --- | --- | --- |
| **Level of care** | Hospital | 1.00 |  |  |  |
|  | Non-hospital formal medical | 0.81 | 0.49 | 1.35 | 0.426 |
|  | CHW | 0.65 | 0.25 | 1.69 | 0.376 |
|  | Pharmacy | 0.06 | 0.01 | 0.27 | <0.001 |
|  | Other | 0.18 | 0.08 | 0.42 | <0.001 |
|  | No care sought | 0.07 | 0.04 | 0.13 | <0.001 |
| **Malaria endemicity** | Low stable transmission | 1.00 |  |  |  |
|  | Moderate stable transmission | 1.62 | 1.06 | 2.46 | 0.024 |
|  | High stable transmission | 5.49 | 1.69 | 17.82 | 0.005 |
| **Child's age (in months)** | 0 - 5 | 0.85 | 0.42 | 1.73 | 0.659 |
|  | 6 - 11 | 1.00 |  |  |  |
|  | 12 - 23 | 1.33 | 0.77 | 2.32 | 0.308 |
|  | 24 - 35 | 1.90 | 1.08 | 3.32 | 0.025 |
|  | 36 - 47 | 1.43 | 0.78 | 2.59 | 0.245 |
|  | 48 - 59 | 2.56 | 1.37 | 4.80 | 0.003 |
| **Child's sex** | Male | 1.00 |  |  |  |
|  | Female | 1.04 | 0.76 | 1.44 | 0.788 |
| **Maternal age (in years)** | 15 -24 | 1.00 |  |  |  |
|  | 25 - 29 | 1.01 | 0.66 | 1.55 | 0.966 |
|  | 30 - 34 | 1.10 | 0.70 | 1.74 | 0.680 |
|  | 35 - 39 | 0.88 | 0.52 | 1.47 | 0.619 |
|  | 40 - 49 | 0.67 | 0.34 | 1.33 | 0.248 |
| **Maternal education** | None | 1.00 |  |  |  |
|  | Primary attendance | 1.09 | 0.72 | 1.64 | 0.697 |
|  | At least secondary attendance | 1.08 | 0.61 | 1.91 | 0.788 |
| **Household wealth index** | Poorest | 1.00 |  |  |  |
|  | Second | 1.00 | 0.61 | 1.64 | 0.997 |
|  | Middle | 1.04 | 0.60 | 1.80 | 0.902 |
|  | Fourth | 1.27 | 0.67 | 2.40 | 0.467 |
|  | Least poor | 1.38 | 0.68 | 2.81 | 0.374 |
| **Total household members** | 1-4 members | 1.00 |  |  |  |
|  | 5-8 members | 0.77 | 0.34 | 1.75 | 0.536 |
|  | 9-12 members | 0.44 | 0.19 | 1.00 | 0.051 |
|  | 13 or more members | 0.59 | 0.27 | 1.29 | 0.183 |
| **Residence** | Urban | 1.00 |  |  |  |
|  | Rural | 1.28 | 0.79 | 2.07 | 0.314 |

Table legend:

AOR based on mixed-effects logistic regression models adjusted for data clustering and confounding covariates. PSUs included in model as random effects. All listed covariates included as fixed categorical effects.

**Tanzania**

**AIDS and Malaria Indicator Survey 2011-2012**

|  |  | **AOR** | **95% CI** | | **pvalue** |
| --- | --- | --- | --- | --- | --- |
| **Level of care** | Hospital | 1.00 |  |  |  |
|  | Non-hospital formal medical | 0.33 | 0.18 | 0.60 | <0.001 |
|  | CHW |  |  |  |  |
|  | Pharmacy | 0.01 | 0.00 | 0.02 | <0.001 |
|  | Other | 0.04 | 0.01 | 0.23 | <0.001 |
|  | No care sought | 0.00 | 0.00 | 0.01 | <0.001 |
| **Malaria endemicity** | No transmission | 0.45 | 0.04 | 4.94 | 0.513 |
|  | Low stable transmission | 1.00 |  |  |  |
|  | Moderate stable transmission | 1.62 | 0.92 | 2.86 | 0.097 |
|  | High stable transmission | 0.76 | 0.33 | 1.75 | 0.517 |
| **Child's age (in months)** | 0 - 5 | 0.21 | 0.09 | 0.52 | 0.001 |
|  | 6 - 11 | 1.00 |  |  |  |
|  | 12 - 23 | 0.99 | 0.59 | 1.67 | 0.964 |
|  | 24 - 35 | 0.94 | 0.54 | 1.65 | 0.836 |
|  | 36 - 47 | 1.02 | 0.55 | 1.88 | 0.953 |
|  | 48 - 59 | 1.16 | 0.57 | 2.35 | 0.678 |
| **Child's sex** | Male | 1.00 |  |  |  |
|  | Female | 1.04 | 0.72 | 1.49 | 0.839 |
| **Maternal age (in years)** | 15 -24 | 1.00 |  |  |  |
|  | 25 - 29 | 1.23 | 0.77 | 1.99 | 0.387 |
|  | 30 - 34 | 0.82 | 0.47 | 1.42 | 0.481 |
|  | 35 - 39 | 0.73 | 0.40 | 1.33 | 0.305 |
|  | 40 - 49 | 0.61 | 0.30 | 1.24 | 0.172 |
| **Maternal education** | None | 1.00 |  |  |  |
|  | Primary attendance | 0.72 | 0.44 | 1.16 | 0.175 |
|  | At least secondary attendance | 1.40 | 0.68 | 2.86 | 0.360 |
| **Household wealth index** | Poorest | 1.00 |  |  |  |
|  | Second | 0.53 | 0.29 | 0.98 | 0.043 |
|  | Middle | 1.11 | 0.62 | 1.99 | 0.731 |
|  | Fourth | 1.35 | 0.76 | 2.42 | 0.309 |
|  | Least poor | 1.84 | 0.84 | 4.04 | 0.127 |
| **Total household members** | 1-4 members | 1.00 |  |  |  |
|  | 5-8 members | 0.70 | 0.44 | 1.12 | 0.137 |
|  | 9-12 members | 1.02 | 0.56 | 1.85 | 0.951 |
|  | 13 or more members | 0.95 | 0.44 | 2.02 | 0.886 |
| **Residence** | Urban | 1.00 |  |  |  |
|  | Rural | 0.21 | 0.10 | 0.42 | <0.001 |

Table legend:

AOR based on mixed-effects logistic regression models adjusted for data clustering and confounding covariates. PSUs included in model as random effects. All listed covariates included as fixed categorical effects.

**Uganda**

**Demographic and Health Survey 2011**

| \|  \|  \| **AOR** \| **95% CI** \| \| **pvalue** \| \| --- \| --- \| --- \| --- \| --- \| --- \| \| **Level of care** \| Hospital \| 1.00 \|  \|  \|  \| \|  \| Non-hospital formal medical \| 2.10 \| 1.67 \| 2.64 \| <0.001 \| \|  \| CHW \| 0.65 \| 0.29 \| 1.47 \| 0.303 \| \|  \| Pharmacy \| 0.44 \| 0.19 \| 1.01 \| 0.052 \| \|  \| Other \| 0.35 \| 0.18 \| 0.68 \| 0.002 \| \|  \| No care sought \| 0.12 \| 0.07 \| 0.20 \| <0.001 \| \| **Malaria endemicity** \| No transmission \| 46295.73 \| 6.70E-189 \| 3.20E+197 \| 0.962 \| \|  \| Low stable transmission \| 1.00 \|  \|  \|  \| \|  \| Moderate stable transmission \| 133727.90 \| 1.90E-188 \| 9.20E+197 \| 0.958 \| \|  \| High stable transmission \| 95144.07 \| 1.40E-188 \| 6.50E+197 \| 0.960 \| \| **Child's age (in months)** \| 0 - 5 \| 0.80 \| 0.50 \| 1.29 \| 0.354 \| \|  \| 6 - 11 \| 1.00 \|  \|  \|  \| \|  \| 12 - 23 \| 1.27 \| 0.91 \| 1.77 \| 0.154 \| \|  \| 24 - 35 \| 1.20 \| 0.86 \| 1.68 \| 0.288 \| \|  \| 36 - 47 \| 0.94 \| 0.66 \| 1.35 \| 0.744 \| \|  \| 48 - 59 \| 1.04 \| 0.72 \| 1.50 \| 0.840 \| \| **Child's sex** \| Male \| 1.00 \|  \|  \|  \| \|  \| Female \| 0.99 \| 0.81 \| 1.20 \| 0.893 \| \| **Maternal age (in years)** \| 15 -24 \| 1.00 \|  \|  \|  \| \|  \| 25 - 29 \| 0.89 \| 0.69 \| 1.15 \| 0.388 \| \|  \| 30 - 34 \| 0.89 \| 0.66 \| 1.21 \| 0.459 \| \|  \| 35 - 39 \| 0.65 \| 0.46 \| 0.92 \| 0.015 \| \|  \| 40 - 49 \| 0.70 \| 0.46 \| 1.09 \| 0.114 \| \| **Maternal education** \| None \| 1.00 \|  \|  \|  \| \|  \| Primary attendance \| 1.15 \| 0.84 \| 1.57 \| 0.387 \| \|  \| At least secondary attendance \| 1.34 \| 0.91 \| 1.99 \| 0.141 \| \| **Household wealth index** \| Poorest \| 1.00 \|  \|  \|  \| \|  \| Second \| 0.72 \| 0.53 \| 0.99 \| 0.043 \| \|  \| Middle \| 0.88 \| 0.63 \| 1.23 \| 0.451 \| \|  \| Fourth \| 0.99 \| 0.70 \| 1.41 \| 0.964 \| \|  \| Least poor \| 1.77 \| 1.18 \| 2.66 \| 0.006 \| \| **Total household members** \| 1-4 members \| 1.00 \|  \|  \|  \| \|  \| 5-8 members \| 0.97 \| 0.75 \| 1.24 \| 0.793 \| \|  \| 9-12 members \| 1.07 \| 0.78 \| 1.48 \| 0.665 \| \|  \| 13 or more members \| 0.78 \| 0.38 \| 1.59 \| 0.492 \| \| **Residence** \| Urban \| 1.00 \|  \|  \|  \| \|  \| Rural \| 0.42 \| 0.29 \| 0.60 \| <0.001 \| |  |  |  | |  |
| --- | --- | --- | --- | --- | --- | --- | --- | --- | --- | --- | --- | --- | --- | --- | --- | --- | --- | --- | --- | --- | --- | --- | --- | --- | --- | --- | --- | --- | --- | --- | --- | --- | --- | --- | --- | --- | --- | --- | --- | --- | --- | --- | --- | --- | --- | --- | --- | --- | --- | --- | --- | --- | --- | --- | --- | --- | --- | --- | --- | --- | --- | --- | --- | --- | --- | --- | --- | --- | --- | --- | --- | --- | --- | --- | --- | --- | --- | --- | --- | --- | --- | --- | --- | --- | --- | --- | --- | --- | --- | --- | --- | --- | --- | --- | --- | --- | --- | --- | --- | --- | --- | --- | --- | --- | --- | --- | --- | --- | --- | --- | --- | --- | --- | --- | --- | --- | --- | --- | --- | --- | --- | --- | --- | --- | --- | --- | --- | --- | --- | --- | --- | --- | --- | --- | --- | --- | --- | --- | --- | --- | --- | --- | --- | --- | --- | --- | --- | --- | --- | --- | --- | --- | --- | --- | --- | --- | --- | --- | --- | --- | --- | --- | --- | --- | --- | --- | --- | --- | --- | --- | --- | --- | --- | --- | --- | --- | --- | --- | --- | --- | --- | --- | --- | --- | --- | --- | --- | --- | --- | --- | --- | --- | --- | --- | --- | --- | --- | --- | --- | --- | --- | --- | --- | --- | --- | --- | --- | --- | --- | --- | --- | --- | --- | --- | --- | --- | --- | --- | --- | --- | --- | --- | --- | --- | --- | --- | --- | --- | --- | --- | --- | --- | --- |
|  |  |  |  |  |  |
| Table legend:  AOR based on mixed-effects logistic regression models adjusted for data clustering and confounding covariates. PSUs included in model as random effects. All listed covariates included as fixed categorical effects. |  |  |  |  |  |

**Zimbabwe**

**Demographic and Health Survey 2010-2011**

|  |  | **AOR** | **95% CI** | | **pvalue** |
| --- | --- | --- | --- | --- | --- |
| **Level of care** | Hospital | 1.00 |  |  |  |
|  | Non-hospital formal medical | 0.34 | 0.13 | 0.90 | 0.030 |
| **Malaria endemicity** | Low stable transmission | 1.00 |  |  |  |
|  | Moderate stable transmission | 1.74 | 0.75 | 4.03 | 0.196 |
| **Child's age (in months)** | 0 - 5 | 1.53 | 0.34 | 6.97 | 0.583 |
|  | 6 - 11 | 1.00 |  |  |  |
|  | 12 - 23 | 0.95 | 0.27 | 3.35 | 0.941 |
|  | 24 - 35 | 1.38 | 0.39 | 4.86 | 0.614 |
|  | 36 - 47 | 0.83 | 0.20 | 3.41 | 0.799 |
|  | 48 - 59 | 2.43 | 0.67 | 8.82 | 0.176 |
| **Child's sex** | Male | 1.00 |  |  |  |
|  | Female | 0.68 | 0.32 | 1.45 | 0.314 |
| **Maternal age (in years)** | 15 -24 | 1.00 |  |  |  |
|  | 25 - 29 | 0.62 | 0.22 | 1.71 | 0.356 |
|  | 30 - 34 | 1.18 | 0.39 | 3.54 | 0.773 |
|  | 35 - 39 | 2.93 | 1.01 | 8.53 | 0.049 |
|  | 40 - 49 | 0.00 | 0.00 | . | 0.990 |
| **Maternal education** | Primary or no attendance | 1.00 |  |  |  |
|  | At least secondary attendance | 1.62 | 0.69 | 3.82 | 0.268 |
| **Household wealth index** | Poorest | 1.00 |  |  |  |
|  | Second | 2.99 | 1.12 | 8.02 | 0.029 |
|  | Middle | 0.80 | 0.24 | 2.67 | 0.716 |
|  | Fourth | 1.00 | 0.22 | 4.46 | 0.999 |
|  | Least poor | 9.41 | 1.74 | 50.87 | 0.009 |
| **Total household members** | 1-4 members | 1.00 |  |  |  |
|  | 5-8 members | 0.94 | 0.40 | 2.19 | 0.887 |
|  | 9 or more members | 0.78 | 0.21 | 2.86 | 0.706 |
| **Residence** | Urban | 1.00 |  |  |  |
|  | Rural | 8.35 | 1.65 | 42.35 | 0.010 |

Table legend:

AOR based on mixed-effects logistic regression models adjusted for data clustering and confounding covariates. PSUs included in model as random effects. All listed covariates included as fixed categorical effects.
